# Clinical Assessment and Management of Spondyloarthritides in the Middle East: A Multinational Investigation

**DOI:** 10.1155/2015/178750

**Published:** 2015-12-17

**Authors:** Mohammed Hammoudeh, Hanan Al Rayes, Adel Alawadhi, Kamel Gado, Khalid Shirazy, Atul Deodhar

**Affiliations:** ^1^Hamad Medical Corporation, Department of Medicine, Doha, Qatar; ^2^Prince Sultan Military Medical Center, P.O. Box 5004, Riyadh 11422, Saudi Arabia; ^3^Department of Medicine, Faculty of Medicine, Kuwait University, P.O. Box 24923, 13110 Al-Safat, Kuwait; ^4^Rheumatology and Immunology Unit, Internal Medicine Department, Cairo University, Cairo 11562, Egypt; ^5^Pfizer Inc. Africa & Middle East, Dubai, UAE; ^6^Oregon Health & Science University, Portland, OR 97239, USA

## Abstract

Data on spondyloarthritis (SpA) from the Middle East are sparse and the management of these diseases in this area of the world faces a number of challenges, including the relevant resources to enable early diagnosis and referral and sufficient funds to aid the most appropriate treatment strategy. The objective was to report on the characteristics, disease burden, and treatment of SpA in the Middle East region and to highlight where management strategies could be improved, with the overall aim of achieving better patient outcomes. This multicenter, observational, cross-sectional study collected demographic, clinical, laboratory, and treatment data on 169 consecutive SpA patients at four centers (Egypt, Kuwait, Qatar, and Saudi Arabia). The data collected presents the average time from symptom onset to diagnosis along with the presence of comorbidities in the region and comparisons between treatment with NSAIDs and biologics. In the absence of regional registries of SpA patients, the data presented here provide a rare snapshot of the characteristics, disease burden, and treatment of these patients, highlighting the management challenges in the region.

## 1. Introduction

The spondyloarthritides (SpA) are a group of inflammatory rheumatic disorders related by clinical symptoms and genetic predisposition. They include ankylosing spondylitis (AS), psoriatic arthritis (PsA), reactive arthritis, inflammatory bowel disease- (IBD-) related SpA, and undifferentiated SpA [1]. They share a number of axial manifestations (sacroiliitis and spinal involvement), peripheral musculoskeletal abnormalities (arthritis, dactylitis, and enthesitis), extra-articular symptoms (e.g., uveitis), and genetic characteristics (HLA-B27 and familial clustering) [1]. Classification criteria have been developed for peripheral and axial SpA [2–4].

The prevalence of SpA shows considerable differences among ethnic groups and populations and depends on the prevalence of the HLA-B27 gene in the population studied [5]. Estimates of the prevalence of SpA vary from 0.31% in France and 0.49% in Greece to 0.9% in the USA [1, 6, 7]. There are currently very few data available on the prevalence of SpA in the Middle East. It has been reported that the prevalence of SpA in the Middle East is low; a study from Iran estimated a prevalence of 0.23% [8], and an earlier study in North Pakistan reported a prevalence of 0.1% [9]. Part of the variation in prevalence is probably explained by differences in quality and bias of the methodological approaches used in epidemiological studies [5].

The variation in incidence and prevalence of SpA can also partly be attributed to geographic variation in the prevalence of HLA-B27. The HLA-B27 antigen is associated with SpA [3, 4], particularly with AS [5], and is one of the factors predicting radiographic progression [10, 11]. However, in general, the association of HLA-B27 with AS appears to be weaker in most Arab countries than in Western European populations [5, 12, 13]. Associations of between 25% and 75% have been reported in Middle Eastern countries, compared with >90% in Western Europe [12]. For example, one study found a prevalence of HLA-B27 of 2.4% among healthy Jordanians and of 71% and 73% among AS patients in Jordan and Qatar, respectively [14]. However, a stronger association has been reported in Iran, with a prevalence of approximately 2.5% in the general population and 92% (23/25) in AS patients [15].

Early detection of axial SpA before radiographic sacroiliitis is present can be challenging in settings where access to specialist MRI facilities is limited and awareness among patients and physicians is low [13]. An approach to screening and referral for possible axial SpA in patients with chronic back pain, based on the presence of inflammatory back pain or HLA-B27 positivity and without the need for imaging, has been proposed [10].

International and regional guidelines recommend the use of TNFi therapy in SpA patients with persistently high disease activity despite treatment with two nonsteroidal anti-inflammatory drugs (NSAIDs) for at least 2 weeks each [16, 17]. However, widespread use of TNFi agents in the Middle East may be hampered by their high cost and increased risk of infections such as tuberculosis (TB) [13]. The risk of TB can be reduced by screening for latent TB infection and treating accordingly [13].

A study by Tayel et al. published in 2012 characterized the functional status, treatment use, and quality of life in a cohort of 75 Egyptian SpA patients; in this study (64% had AS, of whom 59% were positive for HLA-B27) [18], 84% of patients were male, and 5% had uveitis. Almost half (47%) of the patients were unemployed, and 14% were receiving TNFi therapy [18].

The current paper reports on a cross-sectional observational study that expands on the Egyptian study by Tayel et al. [18], and we herein report the current status of SpA and its management across a wider region. To our knowledge, this is the first study of this type to be conducted in the Middle East.

## 2. Methods

### 2.1. Study Design and Participants

This was a multicenter, observational, cross-sectional study conducted in four centers in four countries (Egypt, Kuwait, Qatar, and Saudi Arabia). Participating physicians recorded the following data using a specially designed form for all their patients sequentially seen between March 22, 2013, and July 9, 2013, with SpA (AS, PsA, IBD-related SpA, reactive arthritis, and undifferentiated SpA). The protocol for the research project was approved by a suitably constituted ethics committee of the institution within which work was undertaken, conforming to the provisions of the World Medical Association's Declaration of Helsinki and patient anonymity has been preserved:

- demographics—age, gender, ethnicity, country of birth, smoking status, and family history of rheumatologic disease;
- diagnosis—type of SpA, date of onset of symptoms, date of diagnosis, disease pattern (predominantly axial, predominantly peripheral, and mixed axial and peripheral);
- employment status (full-time, part-time by choice, part-time as a result of disease status, unemployed by choice, and unemployed as a result of disease status);
- laboratory features—HLA-B27 status, erythrocyte sedimentation rate (ESR), and C-reactive protein (CRP);
- radiographic data (AS patients)—radiographic sacroiliitis, MRI features of sacroiliitis;
- clinical disease measures (AS patients)—Ankylosing Spondylitis Disease Activity Score (ASDAS), Bath Ankylosing Spondylitis Disease Activity Index (BASDAI), and Bath Ankylosing Spondylitis Functional Index (BASFI);
- patient-reported outcome measures (AS patients)—Ankylosing Spondylitis Quality of Life (ASQoL) score;
- comorbidities data—uveitis, IBD, psoriasis, cardiovascular disease, hepatitis B and C, tuberculosis, HIV, and other complications;
- treatment—type of treatment (NSAIDs, disease-modifying antirheumatic drugs (DMARDs), and biologics), duration of treatment, and data on eligibility for biologic but either contraindicated or unaffordable.

## 3. Results

### 3.1. Demographic and Disease Characteristics

Data were collected on a total of 169 consecutive patients, 59% were male and 64% were of Arab race, and the mean age was 41.4 years (SD 10.5).  Table 1 shows the demographic characteristics of the patients. In summary, two-thirds of the patients were nonsmokers, and 25% had a family history of rheumatologic disease. Just over half of the patients were in full-time employment; 10% were working part-time as a result of their disease status, and 5% were unemployed for the same reason.

Patients' clinical and disease characteristics, comorbidities, and treatment are summarized in Table 2. Forty-four percent of all patients had AS and 36% had PsA, whilst smaller proportions had IBD-related SpA, reactive arthritis, or undifferentiated SpA. The average age at onset of symptoms was 32.3 years (SD 9.9), and the average age at diagnosis was 34.9 years (SD 9.8). The mean time delay in diagnosis from the onset of symptoms was 2.8 years (SD 4.2). This delay was longest for AS patients, at 4.9 years (SD 5.1), compared with those with PsA at 1.1 years (SD 2.2), IBD-related SpA at 1.6 years (SD 2.7), reactive arthritis at 0.2 years (SD 0.2), and undifferentiated SpA at 1.3 years (SD 1.9). A high proportion of AS patients (74%) had a disease pattern with predominantly axial involvement, whereas most non-AS patients had predominantly peripheral involvement (values ranging between 58% and 100%). Radiographic data show that 82% of AS patients had radiographic sacroiliitis at the time of enrolment in the study. For 35% of AS patients, the diagnosis was made by the presence of sacroiliitis in MRI and, of these patients, 30% had not previously met the New York Criteria for radiographic sacroiliitis.

Of all patients, 70/169 (41%) had unknown HLA-B27 status. Of the 99 patients whose HLA-B27 status was known, 65% (64/169) were positive. Of the 73 AS patients with known HLA-B27 status, 51 (70%) were HLA-B27 positive; of the 26 patients with all other forms of SpA with known HLA-B27 status, 13 (50%) were HLA-B27 positive. The mean ESR was 34.5 mm/h (SD 17.8, range 2.0–96.0), and the mean CRP was 19.5 mg/L (SD 25.1, range 0.6–197). Among AS patients, mean ASDAS was 2.2 (SD 0.9, range 0.8–3.9), mean BASDAI was 3.3 (SD 1.6, range 0.4–8.0), and mean BASFI was 4.1 (SD 1.7, range 0.6–7.6). AS patients reported an average ASQoL score of 9.2 (SD 4.3, range 0.0–18.0).

With regard to comorbidities, 15.4% of all patients (25.7% of AS patients) had uveitis, 4.1% had hepatitis C, 1.8% had cardiovascular disease, and 0.6% had hepatitis B; no patients had tuberculosis or HIV (Table 2).

During the study period, most patients (67%) were being treated with NSAIDs (Table 2). In addition, 21% of patients were being treated with methotrexate, 14% with sulfasalazine, and 4% with leflunomide. No patients were treated with hydroxychloroquine. Thirty-four percent of patients were currently receiving biologics that included adalimumab (11%), certolizumab (0.6%), etanercept (15%), golimumab (1%), infliximab (5%), and ustekinumab (2%). Nineteen percent of patients were reported to be eligible for biologics but not receiving them due to financial limitations.

## 4. Discussion

Compared with the previously described Egyptian sample [18], the percentage of male patients in this sample from four Middle Eastern countries was much lower (59% versus 84%) and the mean age was slightly higher (41.4 versus 37.4 years). The reported male to female ratio among SpA patients worldwide shows great variation, from 0.5 : 1 in the USA and 0.72 : 1 in Turkey to 5.5 : 1 in Greece and 7 : 1 in the Azores [5].

### 4.1. Evidence of Spondyloarthropathies across the Middle East

The proportions of patients with AS, PsA, and reactive arthritis were similar to those in the Egyptian study (44% versus 45%, 36% versus 31%, and 3% versus 3%, resp.), but more patients in the current study had diagnoses of IBD-related SpA (6% versus 1%) and undifferentiated SpA (11% versus 0%) [18]. The authors of the Egyptian study speculated that the absence of undifferentiated SpA patients may have been due to the long delay between symptom onset and diagnosis, allowing specific diagnoses to emerge, or simply due to underdiagnosis of undifferentiated SpA [18]. In a Spanish registry including 1379 SpA patients, the proportion with AS was higher (61%) and the proportion with PsA was lower (21%) in comparison to the Egyptian study and the current study, while only about 1% had reactive arthritis and 15% had undifferentiated SpA [19].

The proportion of AS patients who were positive for HLA-B27 was higher in the present study than in the Egyptian study (69% versus 59%) [18]. However, this is within the 25–75% range reported for Middle Eastern countries, compared with >90% in Western Europe [12].

### 4.2. Comorbidities

Comorbidities are common in SpA. In one study in Belgium, almost half of patients with AS had extra-articular manifestations, the most common being uveitis (seen in 27% of patients) [20]. Other comorbidities included psoriasis, Crohn's disease, and ulcerative colitis [20]. In a study of AS patients in Norway, cardiovascular disease was the most frequent cause of death [21]. Delay in diagnosis and higher disease activity (as measured by CRP) were independent predictors of mortality [21]. There is some evidence that uveitis is more common in AS patients who are positive for HLA-B27, while psoriasis and IBD are more frequent in HLA-B27-negative patients [5, 22].

### 4.3. Uveitis

In the current study uveitis was reported three times more often than in the Egyptian study (15% versus 5%) [18], with a similar rate to that seen in the Spanish study (16%) [19]. The rate of uveitis in patients with a diagnosis of AS was slightly higher than that found in the Spanish study (26% versus 22%). The prevalence of other comorbidities was comparable between the current study and the Spanish study (psoriasis 33% versus 25%; IBD 5% versus 4%; and cardiovascular disease 1.8% versus 1.2%) [19].

Disease severity appeared to be fairly similar to the Egyptian study findings, with a similar mean ESR (34.5 versus 36.8 mm/h) and slightly lower mean BASDAI (3.3 versus 4.2) and BASFI (4.1 versus 5.1) scores [18]. The mean ESR in the Spanish study was lower at 18.5 mm/h, while the mean BASDAI and BASFI scores in AS patients were comparable at 4.1 and 3.6 [19]. About 15% of patients were either working part-time or unemployed as a result of their disease status.

### 4.4. Delay of Diagnosis

The mean delay between onset of symptoms and diagnosis of AS was almost 5 years, while the delay was less than 2 years for the other SpA diagnoses. This delay is shorter than those reported in the Spanish registry (8 years) [23] and in various studies in Europe, Iraq, and Burkina Faso, West Africa [13]. Diagnosis of AS has traditionally been made by the presence of radiographic sacroiliitis; however, this can take several years to evolve [10, 13]. The presence of inflammation on sacroiliac joint MRI has a high sensitivity and specificity [10], but this can be challenging in the Middle East region [13]. Only 35% of AS diagnoses were made by MRI in the current study. Clinical symptoms of inflammatory back pain in patients presenting with low back pain increase the likelihood of AS [10]. HLA-B27 positivity increases the likelihood still further, but this is less useful in populations with low association between HLA-B27 and AS; the 70% positivity rate in the present study suggests that this could have a role in the diagnosis of AS. Clinicians in the region need to take all these factors into account when assessing patients with chronic low back pain.

### 4.5. Utilisation of Different Treatment Options

The proportion of SpA patients treated with NSAIDs, DMARDs, and biologics were not dissimilar to those seen in the Spanish sample [19]. Most patients in this study were being treated with NSAIDs, with many also taking methotrexate or (to a lesser extent) sulfasalazine. Most methotrexate use (81%) was in patients with a diagnosis of PsA, while sulfasalazine was most commonly used by patients with AS (39% of use) or IBD-related SpA (30%). Thirty-four percent of patients were receiving biologic drugs, with the highest biologic use amongst patients with PsA (53% of use) and AS (35%). Almost a fifth of patients were assessed as needing biologics but not receiving them due to financial constraints; a similar finding was reported by Tayel et al. in Egypt [18].

### 4.6. Guidelines and Recommendations

Guidelines for the treatment of patients with SpA in the Middle East are lacking. The Pan Arab Rheumatology Society has published recommendations for the use of biological agents for the treatment of rheumatic diseases, which include AS and PsA [17]. It is likely that most clinicians in the region follow the ASAS/EULAR recommendations, but the cost of biologics means that not all eligible patients receive them. The percentage of patients using biologics in this study does not reflect the actual number of patients using these agents in this area of the world because the patients were recruited from selected university and government hospital clinics where the biologics are available at no cost to the patients or at subsidized prices. The high cost of biologics and unavailability of funds to cover these drugs in many government hospitals and the low income of large percentages of the population in the Middle East make the management of patients with spondyloarthritis in this part of the world less than optimum.

### 4.7. Infection Rates

The increased risk of TB associated with TNFi agents also presents a potential barrier to their use. Chest X-rays and tuberculin skin test and/or an interferon-gamma release assay should be used to screen TNFi candidates for latent TB infection and prophylactic anti-TB therapy initiated in patients with a high suspicion of infection [13, 17].

## 5. Conclusions

In the absence of regional registries of SpA patients, the data presented here provide a rare snapshot of the characteristics, disease burden, and treatment of SpA patients in the Middle East. The future challenge is for more centers in more countries to collaborate to include larger patient numbers and extend the available data across the Middle East region.

## Figures and Tables

**Table 1:** Demographic characteristics of *patients* (*n* = 169).

Characteristic	Number (%)
Gender	
Male	99 (58.6)
Female	70 (41.4)
Race	
Arab	108 (63.9)
Asian	43 (25.4)
White	7 (4.2)
African	2 (1.2)
Other or mixed	9 (5.3)
Smoking status	
Nonsmokers	112 (66.3)
Current smokers	46 (27.2)
Previous smokers	9 (5.3)
Not known	2 (1.2)
Employment status	
Full-time	91 (53.8)
Part-time as a result of the disease status	17 (10.1)
Part-time by choice	7 (4.1)
Unemployed as a result of disease status	8 (4.7)
Unemployed by choice	45 (26.7)
Not known	1 (0.6)
Family history of rheumatologic disease	
Yes	43 (25.4)
No	119 (70.4)
Unknown	7 (4.2)

**Table 2:** Summary of clinical and disease characteristics, comorbidities, and treatment of patients, overall and by diagnosis.

Characteristic	Number (%)					
	All patients (*n* = 169)	AS patients (*n* = 74)	PsA patients (*n* = 61)	IBD-related SpA (*n* = 10)	Reactive arthritis (*n* = 5)	Undifferentiated SpA (*n* = 19)
Diagnosis						
Ankylosing spondylitis	74 (43.8)	—	—	—	—	—
Psoriatic arthritis	61 (36.2)	—	—	—	—	—
Inflammatory bowel disease-related SpA	10 (5.9)	—	—	—	—	—
Reactive arthritis	5 (2.9)	—	—	—	—	—
Undifferentiated SpA	19 (11.2)	—	—	—	—	—
Disease pattern						
Predominantly axial involvement	61 (36.1)	55 (74.3)	2 (3.3)	1 (10)	0 (0)	3 (15.8)
Predominantly peripheral involvement	66 (39.0)	1 (1.4)	41 (67.2)	8 (80)	5 (100)	11 (57.9)
Axial and peripheral involvement	38 (22.5)	16 (21.6)	16 (26.2)	1 (10)	0 (0)	5 (26.3)
Not known	4 (2.4)	2 (2.7)	2 (3.3)	0 (0)	0 (0)	0 (0)
Radiographic sacroiliitis present						
Yes	—	61 (82.4)	—	—	—	—
No	—	9 (12.2)	—	—	—	—
Unknown	—	4 (5.4)	—	—	—	—
HLA-B27 status						
Positive	64 (37.9)	51 (68.9)	13 (15.3)			
Negative	35 (20.7)	22 (29.7)	13 (15.3)			
Unknown	70 (41.4)	1 (0.1)	69 (72.6)			
Erythrocyte sedimentation rate (0–100 mm/h)^*∗*^	24.9 (14.7)	36.4 (19.1)	32.8 (16.8)			
C-reactive protein (mg/L)^*∗*^	23.2 (14.1)	17.9 (16.8)	20.9 (30.5)			
Comorbidities						
Uveitis	26 (15.4)	19 (25.7)	3 (4.9)	1 (10)	1 (20)	2 (10.5)
Inflammatory bowel disease	9 (5.3)	2 (2.7)	0 (0)	—	1 (20)	0 (0)
Psoriasis	56 (33.1)	1 (1.4)	—	0 (0)	0 (0)	0 (0)
Cardiovascular disease	3 (1.8)	2 (2.7)	1 (1.6)	0 (0)	0 (0)	0 (0)
Hepatitis B	1 (0.6)	0 (0)	1 (1.6)	0 (0)	0 (0)	0 (0)
Hepatitis C	7 (4.1)	5 (6.8)	1 (1.6)	1 (10)	0 (0)	0 (0)
Tuberculosis	0 (0)	0 (0)	0 (0)	0 (0)	0 (0)	0 (0)
Human immunodeficiency virus	0 (0)	0 (0)	0 (0)	0 (0)	0 (0)	0 (0)
Current NSAID/DMARD treatment						
NSAIDs	114 (67.4)	53 (71.6)	40 (65.6)	1 (10)	4 (80)	16 (84.2)
Methotrexate	36 (21.3)	2 (2.7)	29 (47.5)	0 (0)	0 (0)	5 (26.3)
Sulfasalazine	23 (13.6)	9 (12.2)	1 (1.6)	7 (70)	3 (60)	3 (15.8)
Hydroxychloroquine	0 (0)	0 (0)	0 (0)	0 (0)	0 (0)	0 (0)
Leflunomide	7 (4.1)	—	—	—	—	—
Current biologic treatment						
Biologic	57 (33.7)	30 (40.5)	21 (34.4)	2 (20)	0 (0)	4 (21.1)
Adalimumab	19 (11.2)	9 (12.2)	8 (13.1)	2 (20)	0 (0)	0 (0)
Certolizumab	1 (0.6)	1 (1.4)	—	—	—	—
Etanercept	25 (14.8)	15^†^ (20.3)	7 (11.5)	0 (0)	0 (0)	3 (15.8)
Golimumab	2 (1.2)	2 (2.7)	0 (0)	0 (0)	0 (0)	0 (0)
Infliximab	8 (4.7)	4 (5.4)	3 (4.9)	0 (0)	0 (0)	1 (5.3)
Ustekinumab	3 (1.8)	3 (4.1)	0 (0)	0 (0)	0 (0)	0 (0)

^*∗*^Values are a mean (SD); ^†^1 patient was recorded to be receiving etanercept and adalimumab.

AS: ankylosing spondylitis; DMARD: disease-modifying antirheumatic drugs; IBD: inflammatory bowel disease; NSAID: nonsteroidal anti-inflammatory; PsA: psoriatic arthritis; SpA: spondyloarthritides.
